# Strategies for Targeting Neural Circuits: How to Manipulate Neurons Using Virus Vehicles

**DOI:** 10.3389/fncir.2022.882366

**Published:** 2022-04-29

**Authors:** Yuqing Hui, Xuefeng Zheng, Huijie Zhang, Fang Li, Guangyin Yu, Jiong Li, Jifeng Zhang, Xiaobing Gong, Guoqing Guo

**Affiliations:** ^1^Department of Anatomy, Neuroscience Laboratory for Cognitive and Developmental Disorders, Medical College of Jinan University, Guangzhou; ^2^Department of Gastroenterology, The First Affiliated Hospital of Jinan University, Guangzhou, China

**Keywords:** viral vectors, neural circuits, AAV, RV, optogenetics, chemogenetics

## Abstract

Viral strategies are the leading methods for mapping neural circuits. Viral vehicles combined with genetic tools provide the possibility to visualize entire functional neural networks and monitor and manipulate neural circuit functions by high-resolution cell type- and projection-specific targeting. Optogenetics and chemogenetics drive brain research forward by exploring causal relationships among different brain regions. Viral strategies offer a fresh perspective for the analysis of the structure-function relationship of the neural circuitry. In this review, we summarize current and emerging viral strategies for targeting neural circuits and focus on adeno-associated virus (AAV) vectors.

## Introduction

Neurons connect with each other to form complex but precise networks, namely neural circuits, which lay the foundation for many brain functions, such as cognition, emotion, learning and memory, and sensory and motor functions. Delineating the fine structure and manipulating the activity of neural circuits are essential for a holistic understanding of complicated brain functions in neuroscience research. This requires highly targeted, efficient, and accurate methods that depend to a large extent on the development and application of virus-tracing technology.

In the early research on neural circuits, a series of tracers were developed: anterograde tracers, such as wheat germ agglutinin (WGA) and phaseolus vulgaris agglutinin (PHA), and retrograde tracers, such as fluorogold (FG), cholera toxin subunit B (CTB), carbocyanine, and WGA. However, these traditional tracers show no cell type selectivity and cannot carry exogenous genes, and most of them do not have the capability of transneuronal trafficking. WGA enables transsynaptic transmission but has no directionality, and gets diluted at synaptic connections, which is not conducive to accurate analysis of neural circuits.

Since Kristensson firstly used herpes simplex virus (HSV) as the pathfinders of viral tracers in the 1980s (Kristensson et al., 1982), various viral vectors have quickly become crucial tools for neural circuit studies, such as AAV, rabies virus (RV), pseudorabies virus (PRV), and canine adenovirus (CAV). Among them, AAV has ideal safety (Biosafety Level-1), low immunogenicity, can be packaged to high titers [10^11^–10^14^ viral genomes(vg)/mL] (Grieger et al., 2006; Lock et al., 2010), and achieves stable and long-term transgene expression in the nervous system (Samulski and Muzyczka, 2014; Bennett et al., 2016). These characteristics make AAVs widely used as the preferred vehicles for gene delivery to the nervous system, regarded as one of the most promising viral vectors for neural circuit research and the treatment of neurological diseases (Weinberg et al., 2013; Santiago-Ortiz and Schaffer, 2016; Sudhakar and Richardson, 2019).

**FIGURE 1:**
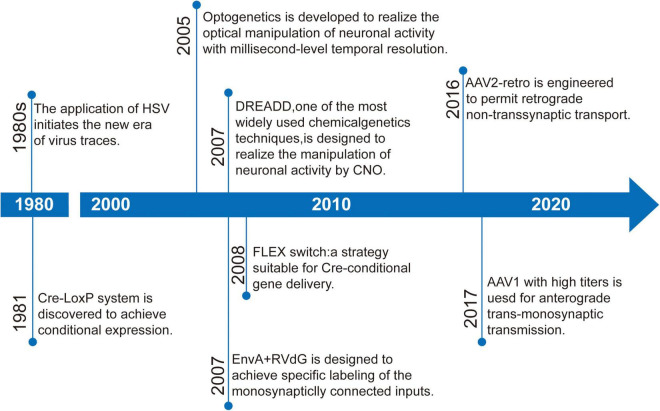
Timeline of some important developments in strategies targeting neural circuits.

Here, we discuss the key advances of viral strategies in neural circuitry (Figure 1), including anterograde and retrograde tracing viruses (Table 1), cell-type and circuit-specific targeting, and monitoring and manipulation of neural circuits.

**TABLE 1:** Properties of commonly used viral vectors in neural circuit tracing.

Type			Virus	Genome size	Vector capacity	Cytotoxicity	Expression efficiency		Transport characteristics
Anterograde	Non-transsynaptic		AAV	∼4.7 kb	∼4.7 kb	Low	Depends on different serotypes	Disadvantage:	Potential retrograde transport
	Trans-synaptic	Trans-monosynaptic	AAV1 with high titer	∼4.7 kb	∼4.7 kb	Low	Low trans-synaptic efficiency	Disadvantage:	Potential retrograde transport
			HSV1-H129-dTK	∼150 kb	∼50 kb	High	Low (due to TK deficiency)	Disadvantage:	Potential retrograde transport
		Trans-multisynaptic	HSV1-H129	∼150 kb	∼50 kb	High	High	Disadvantage:	Potential retrograde transport
			VSV	∼11 kb	∼4 kb	High	High	Disadvantage/advantage:	Bi-directional transport, but pseudotyped VSV with RV-G shows complete retrograde transport
Retrograde	Non-transsynaptic		AAV-retro	∼4.7 kb	∼4.7 kb	Low	Limited subcortical infection	Advantage:	Efficient axon terminal absorption
			CAV-2	∼31 kb	∼30 kb	Moderate	Moderate	Advantage:	Preferentially transduces neuronal axon terminals
			RVdG	∼12 kb	∼3.7 kb	High	High	Advantage:	Complete retrograde transport, efficient infection of axon terminals
	Trans-synaptic	Trans-monosynaptic	EnvA+RVdG	∼12 kb	∼3.7 kb	High	High	Advantage:	Complete retrograde transport, efficient infection of axon terminals
			PRV-dTK	∼142 kb	∼50 kb	High	Low	Disadvantage:	Potential retrograde transport
		Trans-multisynaptic	RV	∼12 kb	∼3.7kb	High	High	Advantage:	Complete retrograde transport, efficient infection of axon terminals
			PRV	∼142 kb	∼50 kb	High	Low	Advantage:	Bartha strain shows complete retrograde transport

*AAV, Adeno-associated virus; HSV1-H129-dTK, Herpes simplex virus type 1; H129, TK-deleted; VSV, Vesicular stomatitis virus; CAV-2, Canine adenovirus 2; RV, Rabies virus; RVdG, Rabies virus, glycoprotein G-deleted; PRV, Pseudorabies virus.*

## Anterograde Tracing: Mapping Output Networks

AAV, belonging to the parvovirus family, is replication-defective and depends on co-infection with a helper virus (such as adenovirus or herpes virus) to accomplish the replication progress. It contains a single-stranded linear DNA genome of approximately 4.7 kilobases (kb) which usually does not integrate into the host genome. The AAV genome mainly encodes two genes, Cap and Rep, and is flanked by two inverted terminal repeats (ITRs,145 bp). The cap open reading frame (ORF) encodes three structural proteins (VP1, VP2 and VP3). These proteins assemble into an icosahedral capsid and participate in cell binding and internalization with the help of an assembly activating protein (AAP; Samulski and Muzyczka, 2014). So that, the cap gene can be modified to control viral tropism and infectivity. The rep ORF encodes four non-structural proteins (Rep78, Rep68, Rep52, and Rep40) that regulate viral genome replication, transcription, virion assembly, and site-specific integration (Pereira et al., 1997). ITRs play an indispensable role in the replication and packaging of the virus as well as the generation of stable episomes; the transgene of interest can be inserted between ITRs to replace the rep and cap genes in recombinant AAV vector systems. After entering the nucleus, the single-stranded genome requires to be transformed into a double-stranded genome, which is considered a key rate-limiting procedure (Fisher et al., 1996) and can be circumvented using self-complementary AAV (scAAV). The scAAV genome contains two complementary transgene sequences connected by a mutated ITR hinge to avoid the requirement of second-strand synthesis, resulting in rapid and significantly efficient expression in the targeted areas. However, the packaging capacity of the vector is reduced to only about half of that of AAV (McCarty et al., 2001; McCarty, 2008). Natural AAV serotypes preferentially exhibit anterograde non-transsynaptic trafficking properties, which can reveal axon projections rather than synaptic connections. In addition, AAV1 at high titers permits anterograde trans-monosynaptic transmission, which reveals the synaptic connections of target cells, and provides more precise interrogation of neural circuits. But its transduction efficiency is highly dependent on virus titer; reducing the titer from 10^13^ GC/ml to 10^11^ GC/ml can make trans-synaptic characteristic disappear (Zingg et al., 2017). AAV1 can achieve effective anterograde trans-synaptic Cre expression through the long-distance spinal-projecting neurons of brain regions (Zingg et al., 2020). However, one defect of AAV1 for anterograde trans-monosynaptic transport is that it can also spread retrogradely at low efficiency (Burger et al., 2004).

Neurotropic viruses have been widely used for transneuronal tracing that is endowed by their replicative life cycle without post-synaptic signal attenuation, which overcomes the transsynaptic “dilution” problem of traditional tracers (Kuypers and Ugolini, 1990; Enquist, 2002; Ugolini, 2008). However, they have high immunogenicity and cytotoxicity, which can lead to the death of infected cells in as little as 3–7 days after administration *in vivo* (Callaway, 2008). Herpes simplex virus type 1 (HSV1) strain H129 and vesicular stomatitis virus (VSV) are often used as anterograde trans-multisynaptic tracers, while RV and PRV are widely used for retrograde trans-multisynaptic labeling (Callaway, 2008). The trans-multisynaptic tracing characteristic of wild-type neuroinvasive viruses causes potential confusion in the identification of the exact input-output architecture (Nassi and Callaway, 2006). Deleting the indispensable genes for replication of neurotropic viruses and complementing these relevant genes in trans with the help of helper virus can achieve trans-monosynaptic transport, which limits input or output tracing only to the next order connections.

HSV-1 belongs to Alphaherpesvirinae, and its ∼150 kb double-stranded (ds) DNA genome provides an extremely large insert capacity for exogenous genes of interest (Li et al., 2020). The HSV-1 strain H129 exhibits predominant trans-polysynaptic trafficking from infected pre- to post-synaptic neurons, making it one of the most promising viral vectors for mapping output circuits (Dum et al., 2009; Lo and Anderson, 2011; McGovern et al., 2012a,b; Yang et al., 2021). Viral thymidine kinase (TK) is indispensable for the replication of H129; therefore, the first anterograde monosynaptic variant (H129-dTK-tdT) has been engineered (Zeng et al., 2017), which is replication-deficient by deleting TK. The helper virus expresses TK to complement the replication of H129-dTK-tdT in trans, and the newly generated progeny virions spread anterogradely to directly connected postsynaptic neurons, where the variant fails to further replicate and transmit due to the lack of TK. Notably, H129 can also undergo temporally delayed retrograde transneuronal transport and labeling (Wojaczynski et al., 2015; Su et al., 2019), which makes the elucidation of the tracing results ambiguous; therefore, rational modification of H129 to minimize or eliminate axon terminal uptake is important for rigorous anterograde tracing. Given the potential retrograde transport properties of AAV1 and H129, ideal anterograde trans-monosynaptic tracer viruses are urgently need to be developed.

VSV is a negative-strand RNA virus that belongs to the Rhabdoviridae family. The VSV glycoprotein (VSV-G) is essential for the binding of the virus to the targeted cell by combining with phosphatidylserine, which is widely expressed on the cell membrane surface and thus makes VSV possess extensive tropism (Lichty et al., 2004; Hastie et al., 2013). Wild-type VSV transmits bidirectionally (Lundh, 1990) determined by the glycoprotein. Pseudotyping VSV with the glycoprotein of lymphocytic choriomeningitis virus (LCMV) endows VSV with the capacity of completely anterograde trans-synaptic transport, whereas pseudotyping VSV with the glycoprotein of RV permits exclusively retrograde trans-synaptic transmission (Beier et al., 2011).

## Retrograde Tracing: Mapping Input Networks

Canine adenovirus-2 (CAV-2) belongs to the Adenoviridae family and can be replication-defective by deleting early region 1 (E1), with a packing capacity of up to 30kb (Junyent and Kremer, 2015). CAV-2 preferentially transduces neuronal axon terminals compared to the soma by binding to the adenovirus receptor (CAR), and thus has the ability of retrograde trafficking (from axon terminals to the soma; Soudais et al., 2001). However, CAV-2 demonstrates limited transgene expression efficiency and exhibits cytotoxicity (Piersanti et al., 2013; Simão et al., 2016).

The engineered variant AAV2-retro provides greater retrograde transport efficiency of up to two orders of magnitude than traditional AAV serotypes. It can exhibit effective retrograde access to projection neurons, and has less immunogenicity (Tervo et al., 2016). These basic characteristics have greatly expanded its role in neural circuit tracing and gene therapy in the central nervous system.

PRV belongs to Alphaherpesvirinae, similar to HSV-1, and is also amenable for the insertion of large exogenous genes, but preferentially spreads retrogradely. One attenuated strain, PRV-Bartha, demonstrates complete retrograde trans-multisynaptic trafficking with reduced cytotoxicity compared to wild-type PRV (Song et al., 2005). A derivative of PRV-Bartha, Ba2001 strian (DeFalco et al., 2001), allows TK expression to accomplish the replication of viral genomes only in Cre-expressing cells and transports to synaptically connected neurons.

RV is a negative-sense, single-stranded RNA virus that is a member of the Rhabdoviridae family, similar to VSV. Unlike viruses possessing a DNA genome, it cannot be used in combination with promoter-specific expression and intracellular genetic manipulation (for example, Cre or Flp recombinase). RV specifically infects neuronal axon terminals, exhibiting complete trans-chemical synaptic transportation without changing neuronal metabolism, although its effective load capacity is limited (only approximately 3.7 kb; Osakada and Callaway, 2013). Rabies glycoprotein (RG) is an envelope protein that facilitates the budding of virions and infection of neurons through axon terminals, but does not affect virus packaging or the transcription and replication of the viral genome, which is regulated by the matrix protein (M; Wickersham et al., 2007). Hence, deleting the RG gene and replacing it with a fluorescent protein gene prevents glycoprotein (G)-deleted rabies virus (RVdG) from transsynaptic spreading (Callaway and Luo, 2015). Pseudotyping RVdG with EnvA (an avian virus envelope protein) enables cell-type-selective infection because EnvA specifically binds to the avian viral receptor (TVA), which is not expressed in the mammalian brain. When TVA and G are co-transfected into particular neuronal populations, EnvA pseudotyped RVdG (EnvA + RVdG) can only infect genetically targeted neurons expressing TVA, and then spread retrogradely from G-expressing starter neurons to directly connected presynaptic neurons. Because direct presynaptic inputs do not express RG, EnvA + RVdG allows specific labeling of the monosynaptically connected inputs as defined neuron subpopulations and cannot achieve further transsynaptic propagation. AAV and lentivirus (LV) are usually used as helper viruses to complement TVA and G; their injection concentration can directly affect the efficiency and specificity of monosynaptically restricted transsynaptic trafficking of EnvA + RVdG (Lavin et al., 2020). Compared to the traditional SAD-B19 (ΔG) strain, the newly developed CVS-N2c (ΔG) rabies virus strain has enhanced neurotropic properties of retrograde transsynaptic trafficking and greatly reduced toxicity (Reardon et al., 2016). Compared with AAV2-retro, CVS-N2c (ΔG) has the same retrograde tracing efficiency, but the more limited diffusion range makes it more suitable for targeting the neuronal inputs to smaller brain regions, and demonstrates a wider tropism of different neuron types and brain regions, especially the subcortical area (Zhu et al., 2020). A new type of “self-inactivating rabies virus” (SiR) allows almost all labeled input neurons to survive for a long time (about several months) and leaves a permanent genetic signature of the traced network before deactivating itself through proteolysis (Ciabatti et al., 2017).

## Cell-Type and Projection Specificity

Neurons of the same cell type in the same brain region may project to different targets executing particular functions and demonstrating specific behavioral characteristics; therefore, only realizing cell type specificity is insufficient to interrogate functionally connected neuronal subpopulations. Targeting specific subpopulations defined not only by cell type but also by connectivity permits high-resolution analysis of neural circuits, which can be achieved through the following methods:

### Capsid Modification

Receptor-mediated endocytosis of viral particles, which is based on the identification between capsid proteins and receptors on the cell surface, is the mechanism of AAV transduction (Bartlett et al., 2000; Pillay et al., 2016). The amino acid sequence of capsids determines cell-type selective tropism and different AAV serotypes; thus, rational capsid modification enables to improve transduction efficiency and alter tropism. AAV2 has relatively high selectivity to neurons and demonstrates limited diffusion in the target region, making it the most widely used serotype and ideal for many neuroscience studies; however, natural AAV capsids with cell type-specific tropism have not yet been discovered. Site-directed mutagenesis of surface-exposed tyrosine residues, for example, tyrosine-to-phenylalanine substitutions, enable the prevention of phosphorylation, capsid ubiquitination, and subsequent proteasome-mediated degradation of AAV2 vectors, consequently leading to increased intracellular trafficking from the cytoplasm to the nucleus and resulting in significantly improved transduction efficiency at lower doses (Zhong et al., 2008a,b; Aslanidi et al., 2013). An AAV2 variant called 7m8, obtained by inserting LALGETTRP after amino acid 587 at AAV2 capsid VP1, demonstrates significant tropism to photoreceptors and retinal pigment epithelium upon intravitreal injection (Dalkara et al., 2013). An AAV6 variant, ShH10, exhibits high tropism (> 90%) for Müller cells through intravitreal injection (Klimczak et al., 2009; Koerber et al., 2009). Furthermore, a novel AAV capsid variant, Olig001, permits significant oligodendrocyte preference (> 95%; Powell et al., 2016).

The blood-brain barrier (BBB) exerts its barrier function to shield the central nervous systems (CNS) from various pathogens and toxins circulating in the blood, however, delivering any viral vectors or drugs to the CNS requires to circumvent this especial barrier function of the BBB when facing with multifocal neurological diseases. By using capsid selection method CREATE (Cre recombinase-based AAV targeted evolution), AAV-PHP.eB, a new BBB-crossing capsid variant, has been selected to enable prominent gene transduction to the CNS with the relatively low vector dose (1 × 10^11^ vg) by peripheral delivery (e.g., retro-orbital or tail vein injection; Chan et al., 2017; Inutsuka et al., 2020). Then the development of AAV.CAP-B10 permits higher neuronal specificity in the CNS and significantly lower liver transduction efficiency after intravenous delivery than AAV-PHP.eB (Goertsen et al., 2022).

### Promoter Specificity

Promoters, the genetic regulatory elements, determine the intensity and cell-type specificity of transgene expression, and the selection of promoters is an important consideration in neuroscience research. The specificity of the promoters is as follows: the cytomegalovirus (CMV) promoter, a ubiquitous promoter, can non-selectively drive transgene expression which is prone to silencing in some tissues over time, and the chicken β-actin/CMV hybrid (CAG) promoter permits stronger and longer expression in neuronal populations (Yaguchi et al., 2013); the human synapsin (hSyn) and neuron-specific enolase (NSE) promoter enable to obtain high levels of neuron-selective expression (Dashkoff et al., 2016); the calcium/calmodulin-dependent kinase II alpha (CaMKIIα) promoter can significantly restrict expression to excitatory neurons (Yaguchi et al., 2013); a synthetic promoter by multimerizing an noradrenergic-specific cis-regulatory element identified in the human dopamine beta-hydroxylase (hDBH) promoter outperforms the natural hDBH promoter in NA neuron-specific transgene expression (Hwang et al., 2001); and the immediate-early gene (IEG) promoter permits labeling of active neurons during particular phases of behavior (Guenthner et al., 2013). Promoter regions are usually large in mammals; attempting to package larger genomes in excess of 5 kb at AAV vectors can bring about dramatically low yield and genome truncation (Dong et al., 2010; Lai et al., 2010; Wu et al., 2010). To address this problem, mini-promoters (MiniPs) have been developed by truncating the partial promoter area (Nathanson et al., 2009; de Leeuw et al., 2016). For example, Ple67 (selective for serotonergic cells), Ple155 (selective for Purkinje cells), and Ple264 (selective for retinal Müller glia) have been proven to be effective for cell type-selective expression (de Leeuw et al., 2016). The challenge of using the cell type-specific promoter within genetically engineered viral vectors can also be transferred to the field of transgenic mice that express Cre or Flp recombinase under the control of different promoters; therefore, recombinase-dependent AAV can facilely achieve any transgene expression (the size is within the packaging range) of cell type specificity. This approach has greatly expanded the application of AAV vectors in neural circuit studies.

### Enhancer Specificity

Enhancers are cis-acting element that could regulate target gene transcription. By activating different sets of enhancers and other distal regulatory elements, a single genome could be transformed into a large number of highly specialized cell subclasses and tissues (Preissl et al., 2018; Klemm et al., 2019). Enhancers, identified by single-cell epigenetic profiling, could therefore provide the molecular genetic tools with the finer grained cell type and region-specific expression programs than promoter-based strategies alone, and the enhancer-based viral tools could also function across species (Vormstein-Schneider et al., 2020; Mich et al., 2021). Additionally, the relatively small size of enhancers makes them easier to package into viral vectors. For instance, the mDlx enhancer is effective for selectively targeting GABAergic interneurons within the telencephalon using AAV (Dimidschstein et al., 2016), the E2 enhancer could restrict gene expression to parvalbumin-expressing cortical interneurons (Vormstein-Schneider et al., 2020).

### The Intersectional Strategy to Achieve Cell Type- And/or Circuit-Specific Gene Delivery

The most commonly used recombinase-based intersectional strategy, in which AAV vectors play an extremely important role, is the combination of double viral vectors. One vector encodes site-specific DNA recombinase (for example, Cre or Flp recombinase), and the other recombinase-dependent vector carries the gene of interest. The transgene can be expressed merely in neuron populations transduced by both vectors, which is called “intersectional targeting”. This strategy can help determine the comprehensive mapping relationship of particular neuron subpopulations receiving the projection of a specific input site or projecting to a specific output site in a targeted brain area.

**FIGURE 2:**
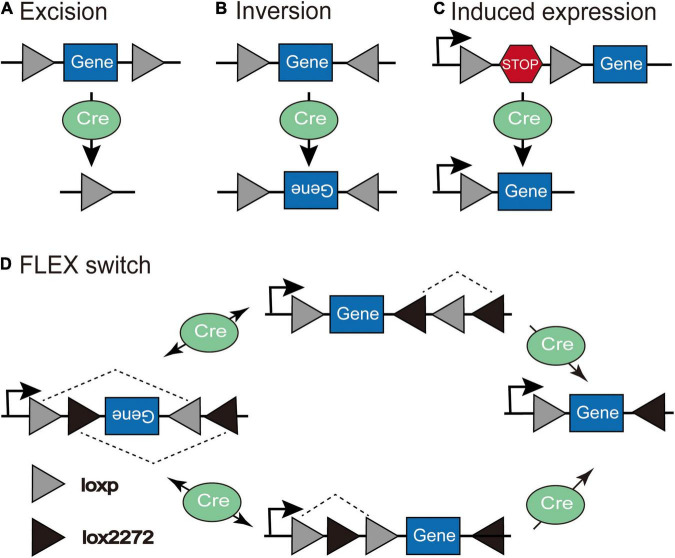
The schematic depiction of the Cre-LoxP system. **(A)** Excision: Cre recombinase cuts the coding sequences between loxP sites in the same orientation. **(B)** Inversion: Cre recombinase leads to the inversion of coding sequences between loxP sites in the opposite orientation. **(C)** Induced expression: Cre recombinase excises the loxP-flanked “STOP” sequence to induce the expression of the transgene. **(D)** In the Flip-Excision (FLEX) switch system, loxP and lox2272 are heterotypic and antiparallel; therefore, after the inversion and subsequent excision, the expression of transgene can be achieved through Cre-mediated inversion of the reverse-constructed coding sequence (Adapted from Atasoy et al., 2008).

The Cre-LoxP system is the most widely utilized conditional expression strategy. Cre recombinase identifies special sequences (lox sites) and drives recombination by deleting coding sequences between lox sites that are in the same direction (Figure 2A) or inducing the reversal of coding sequences between lox sites in the reverse orientation (Figure 2B; Austin et al., 1981; Sauer, 1998; Nagy, 2000). In the “LSL” sequence strategy, the transcription STOP cassette occupying only 1–2 kb is flanked by two identical LoxP sites in the same direction, and inserted between the promoter and gene of interest; thus, Cre recombinase effectively allows the expression of transgene after identifying and excising the loxP-flanked transcriptional stop signal (Figure 2C). However, this method is prone to leak; the “STOP” sequence can be skipped during transcription so that the target gene will be expressed without the action of Cre recombinase. The Flip-Excision (FLEX) switch technology (also called double inverted orientation (DIO) system) employs two pairs of heteromorphic, antiparallel lox-type recombination loci (loxP and lox2272) that are inserted between the coding sequence, which leads to an inversion of the gene sequence and subsequent excision of two different sites, leaving only one of each orthogonal Lox site oriented oppositely to prevent further recombination (Atasoy et al., 2008). In the double-floxed inverted open reading frame, the transgene is packaged in an inverted orientation so that expression can be achieved by flipping into the correct direction in any Cre-positive cells (Figure 2D). Even if transgene expression is not regulated by Cre recombinase and leakage occurs, the reverse-constructed sequence will express a non-functional product, thus making it widely used in the study of neural circuits. A small amount of Cre recombinase is sufficient to permanently activate the gene expression of Cre recombinase-dependent viral vectors (Nagy, 2000). In addition to the Cre-loxP recombinase system, there are Flp-FRT and Dre-Rox recombinase systems with distinct recombinase target sites that do not cross-react mutually (Fenno et al., 2014); therefore, using multiple recombinases intersectionally allows more refined access to specific cell subpopulations.

**FIGURE 3:**
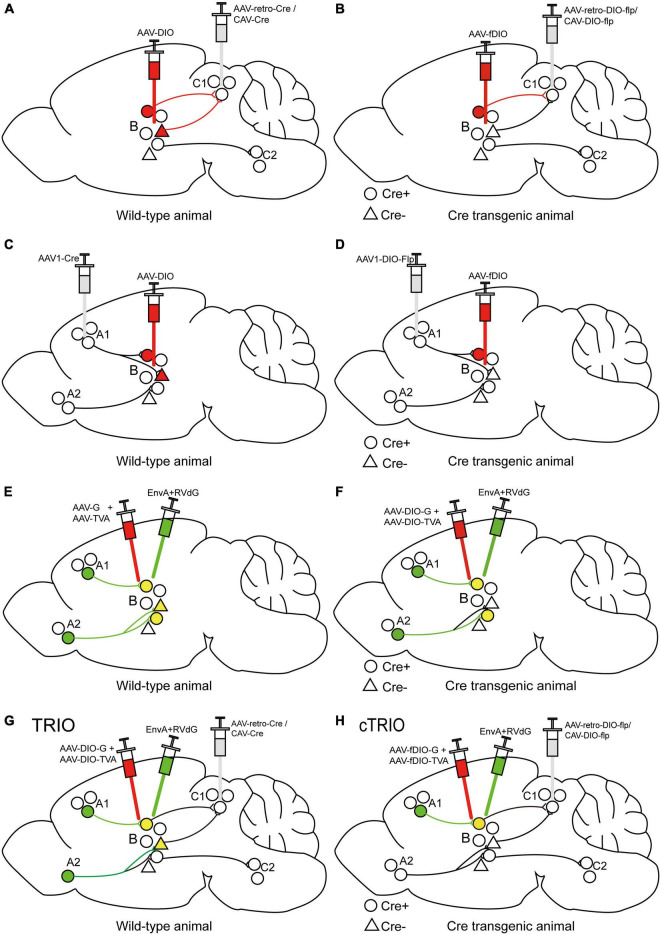
Specific illustrations of labeling neuron subpopulations of interest with cell-type and/or projection specificity. **(A)** The scheme for retrograde tracing to specifically label C1-projecting B neurons. **(B)** The scheme for retrograde tracing combined with Cre transgenic animals to specifically label Cre-expressing neuron subpopulations in region B projecting to region C1. **(C)** The scheme for trans-monosynaptic anterograde tracing to specifically label B neurons receiving inputs from region A1. **(D)** The scheme for trans-monosynaptic anterograde tracing combined with Cre transgenic animals to specifically label Cre-expressing neuron subpopulations in region B receiving inputs from region A1. **(E)** The strategy for trans-monosynaptic retrograde rabies tracing to map direct presynaptic inputs onto B neurons. The starter cells (yellow) represent the co-infection of helper viruses and RVdG. **(F)** The strategy for Cre-dependent, trans-monosynaptic retrograde rabies tracing to map direct presynaptic inputs onto Cre-expressing B neurons. **(G)** The TRIO strategy to map direct presynaptic inputs onto C1-projecting B neurons. **(H)** The cTRIO strategy to map direct presynaptic inputs onto C1-projecting, as well as Cre-expressing B neurons.

The Cre-dependent AAV-DIO virus and retrograde-transporting virus delivering Cre recombinase (such as AAV2-retro-Cre or CAV-Cre) can be injected into the target area and efferent region, respectively, to label output-defined neuron populations in the target field (Figure 3A). This method has been used to label periaqueductal gray (PAG)-projecting medial preoptic area (mPOA) neurons, which are important in regulating anxiety-like behaviors (Zhang et al., 2021), and idendtify ventrolateral orbital cortex (VLO)-projecting submedius thalamic nucleus (Sm) neurons, which are important for the regulation of neuropathic pain-induced anxiodepression (Sheng et al., 2020), and so on. To further achieve cell type-specific neural pathway labeling, we utilize Cre transgenic animals. Retrogradely transported AAV2-retro-DIO-Flp or CAV-DIO-Flp expressing Cre-dependent Flp is injected into a special efferent region of the target area and Flp-dependent AAV-fDIO into the target area of Cre mice to label output-defined and cell type-specific neuron subpopulations (Figure 3B). This method has been used to demonstrate that inhibitory and excitatory inputs from lateral hypothalamus (LH) to the periaqueductal gray (PAG) in the midbrain of mice drive predation and evasion, respectively (Li et al., 2018). A Cre-dependent AAV-DIO virus and an AAV1-Cre virus can be injected into the target and afferent region, respectively, to map the input-defined neuronal population in the target region (Figure 3C). This has been used to recognize the mPOA-recipient PAG neurons, which are important for anxiety-like behaviors (Zhang et al., 2021), and infect VLO neurons that receive inputs of Sm to verify the antianxiodepressive effect of Sm-VLO projection (Sheng et al., 2020). Similarly, to achieve cell type-specific neuron targeting, AAV1-DIO-Flp expressing Cre-dependent Flp is injected into a special afferent region of the target area and Flp-dependent AAV-fDIO into the target area of Cre mice to label input-defined and cell type-specific neuronal subpopulations (Figure 3D), which has been used to label the primary visual cortex (V1)-inputting superior colliculus (SC) glutamatergic or GABAergic neuron subpopulations (Zingg et al., 2017).

Notably, AAV1-Cre can also permit retrograde transport (Aschauer et al., 2013; Tervo et al., 2016), making it not suitable for the interrogation of bidirectional circuits (Zingg et al., 2017), and its directionality can be proved by utilizing traditional retrograde tracers. The defect of these intersectional strategies is that the input or output subpopulations must be spatially isolated from the targeted neuron populations, making it unsuitable for studying local microcircuits. And these strategies require previous knowledge on the studied connectivity (afferent or efferent regions), so are not suitable for unbiased connectivity mapping. In addition, verifying the cell-type specificity of gene expression is necessary in each experimental process.

INTronic Recombinase Sites Enabling Combinatorial Targeting (INTRSECT) system, a versatile single-AAV system, enables intersectional targeting refined cell popolations expressing different recombinases (e.g., C_*on*_/F_*on*_, C_*on*_/F_*off*_ or C_*off*_/F_*on*)_ using multiple-feature Boolean logic (Fenno et al., 2014). The newest tetracycline transactivator (tTA)-driven Recombinase-Guided Intersectional Targeting (tTARGIT) system contains a Flp-dependent tTA “Driver” AAV and a tetracycline response element (TRE)-driven Cre-dependent “Payload” AAV that enables to flexibly target intersectional neural populations (Sabatini et al., 2021).

## Tracing the Relation Between Input and Output (TRIO)/Cell Type Specific TRIO (cTRIO)

Since the trans-monosynaptic retrograde rabies tracing approach has developed (Figures 3E–F; Beier et al., 2015; Callaway and Luo, 2015), the TRIO (tracing the relation between input and output) strategy has appeared subsequently to define starter-cell populations, not only monosynaptic input defined but also output defined by using the combinatorial power of AAV, CAV-2, and RV (EnvA + RVdG) vectors (Figure 3G; Schwarz et al., 2015; An et al., 2020). Briefly, AAV2-retro-Cre or CAV-Cre is injected into a selected output site (C region) of the targeted area (B region), along with AAVs expressing Cre-dependent TVA and G into region B to label starter cells, and the connection from region B to C is determined by the axon terminal uptake of the retrograde-transporting virus. EnvA + RVdG is then injected into region B to specifically infect starter cells and determine direct inputs (A region) of starter cells; the connection from region A to B is identified by RVdG-mediated retrograde trans-monosynaptic tracing with the trans-complement of G. TRIO does not distinguish between starter cell types projecting to a selected output region, which can be achieved by cell-type-specific TRIO (cTRIO). In cTRIO (Beier et al., 2015; Schwarz et al., 2015), AAV2-retro-DIO-Flp or CAV-DIO-Flp expressing Cre-dependent Flp recombinase is injected into a specific C region in Cre transgenic animals, in conjunction with AAVs expressing Flp-dependent TVA and G into region B, to label the specific type of starter cells (Cre^ +^ neurons), followed by EnvA + RVdG (Figure 3H). TRIO/cTRIO technologies enable the identification of monosynaptic inputs of starter cell populations projecting to a specific output target, and have been employed to map the input-output architecture of locus coeruleus noradrenaline (LC-NE) neurons (Schwarz et al., 2015) and ventral tegmental area dopamine (VTA-DA) neurons (Beier et al., 2015).

## Monitoring and Manipulating Neural Circuits

Tracing methods alone can only access the structure of neural circuits and not the function. To address this issue, relevant genetically encoded tools have been developed to monitor, activate, or inhibit neuronal activity, which establishes direct contacts between genetically defined neuron populations and circuit function using cell type- and/or projection-selective gene delivery approaches.

Genetically encoded calcium indicators (GECIs) have been widely used as activity sensors to capture the dynamics of defined neuronal populations by measuring changes of Ca2 + concentration during specific behaviors. The action potential allows the opening of the voltage-gated calcium channel, thus leading to increase in cytoplasmic Ca2 + concentration that can be exhibited through fluorescence intensity of the GECIs (Tian et al., 2009; Grienberger and Konnerth, 2012). Thereby, GECIs display a direct connection between the activity of genetically defined neurons and particular behaviors (such as defense, anxiety, and fear; Besnard et al., 2019; Kennedy et al., 2020; Griessner et al., 2021).

Different categories of microbial genes encoding opsins can achieve light-activated excitatory or inhibitory effects in genetically targeted neurons (Boyden et al., 2005), resulting in specific neuronal control during behavior. For example, channelrhodopsins (ChRs), cation channel opsins, excite neurons by the stimulation of blue light, which causes the inflow of cations to generate depolarization potential. However, halorhodopsin-type Cl^–^ pumps (NpHR) and bacteriorhodopsin-type proton pumps inhibit neurons in response to yellow or green light, respectively, which causes the inflow of Cl^–^ ions or outflow of protons to generate hyperpolarization potential. Optogenetics have brought seminal influence on the interrogation of brain function in neuroscience research (Adamantidis et al., 2015; Shirai and Hayashi-Takagi, 2017). Applying optogenetics to behavioral research has brought multitudinous in-depth insights into anxiety (Kim et al., 2013; Marcinkiewcz et al., 2016; Liu et al., 2020), itch (Mu et al., 2017; Gao et al., 2019; Samineni et al., 2021), reward (Stuber et al., 2011; Han W. et al., 2018) and learning and memory (Qin et al., 2018; Teixeira et al., 2018). For instance, it has been reported that activation of glutamatergic fibers from the basolateral amygdala (BLA) to the nucleus accumbens (NAc) facilitates reward-seeking behaviors (Stuber et al., 2011), and stimulating serotonergic terminals optically in the hippocampal CA1 region from the raphe nuclei is conducive to spatial memory (Teixeira et al., 2018). Opsins can be expressed and distributed throughout the whole neuron; not only can axonal projections of genetically specified neurons be controlled by light delivered directly during behavior, but functional connectivity can also be studied in acute brain slices. Using the sodium channel blocker tetrodotoxin (TTX) in acute brain slices to block polysynaptic responses and the K + channel blocker 4-aminopyridine (4-AP) to allow ChR-driven monosynaptic transmitter release, only monosynaptic responses are optically elicited in putative, direct postsynaptic outputs through light delivery to the presynaptic neurons (Petreanu et al., 2009; Root et al., 2014; Adhikari et al., 2015; Lerner et al., 2015). For example, the optogenetic monosynaptic connectivity method has been used for isolated direct ventromedial prefrontal cortex (mPFC) inputs to the basomedial amygdala (BMA; Adhikari et al., 2015) and striatal-to-midbrain dopamine neurons (Lerner et al., 2015). That is, we can first identify the projection relationship structurally and morphologically by virus traces and then identify whether a functional synaptic connection is established with optogenetics and brain slice electrophysiology.

Designer receptors exclusively activated by designer drugs (DREADD), the most widely used chemogenetic technology, is not activated by acetylcholine or other endogenous neurotransmitters, but is selectively activated or inhibited by the inactive clozapine analog clozapine N-oxide (CNO; Armbruster et al., 2007; Sternson and Roth, 2014). Clozapine, into which CNO rapidly converts *in vivo*, acts on DREADD, namely, mutated human muscarinic receptors (for example, hM3Dq and hM4Di), thereby activating different G protein-coupled pathways to produce the effect of activating or inhibiting neuronal activity (Gomez et al., 2017). Clozapine has high affinity for many receptors and has side effects such as behavioral inhibition (MacLaren et al., 2016). The effect of DREADD on neuronal electrical activity is also unstable due to indirect coupling with ion channels. Engineered ligand-gated ion channels (LGICs) could directly regulate neuronal activity by the small molecule, so a newer chemogenetic platform was developed (Sternson and Roth, 2014). Pharmacologically Selective Actuator Modules (PSAMs), the mutated α7 nAChR ligand binding domains (LBDs), behave as the independent actuator module that could be fused to various ion pore domains (IPDs) with different ionic conductance properties to produce distinct PSAM-IPD chimeric channels (Magnus et al., 2011). For example, chloride-selective PSAM-GlyR and cation-selective PSAM-5HT3 chimeric channels lead to neuronal inhibition or activation, respectively, with the application of the appropriate Pharmacologically Selective Effector Molecule (PSEM) agonists (Magnus et al., 2011). Similar to optogenetics, chemogenetic tools have also been extensively utilized to clarify the role of neural circuits in a particular behavior (Roth, 2016; Vetere et al., 2017; Luo et al., 2020).

Many neuroscience studies require both optogenetic and chemogenetic technologies to disentangle the role of specific neural circuits in the control of particular behaviors. Optogenetics has superior temporal resolution, with an accuracy of millisecond-level temporal control (Boyden et al., 2005; Li et al., 2005). The spatial accuracy can reach the level of single cells or even organelles, but it is difficult for light to penetrate a large brain region, which limits its use in large mammals. Chemogenetics is ideal for neuron populations with a wide spatial distribution but has relatively lower temporal resolution, which makes it more suitable for longer-term manipulation of neuronal circuits. In addition, chemogenetics allows the systemic injection of the “designer drug” to activate or inhibit the target circuit in a non-invasive manner, instead of requiring invasive optical implants to allow the delivery of light to specific brain regions.

The CRISPR/Cas9 system combined with viral vectors can achieve genome editing *in vivo* and activate or repress the activity of endogenous genes in an adjustable manner (Wang et al., 2016; Yim et al., 2020). By delivering the guide RNA and Cas9 enzyme based on the intersectional strategy, CRISPR-mediated target gene editing permits the elucidation of circuit-specific functions (Sarno and Robison, 2018). RNAi technology allows the significant downregulation of gene expression by introducing siRNA or shRNA into target cells, making it a useful tool for research on the functional interrogation of particular genes and RNAi-based therapeutics (Setten et al., 2019).

## Inducible Systems With Precise Temporal Control

### CreER System Induced by Tamoxifen

**FIGURE 4:**
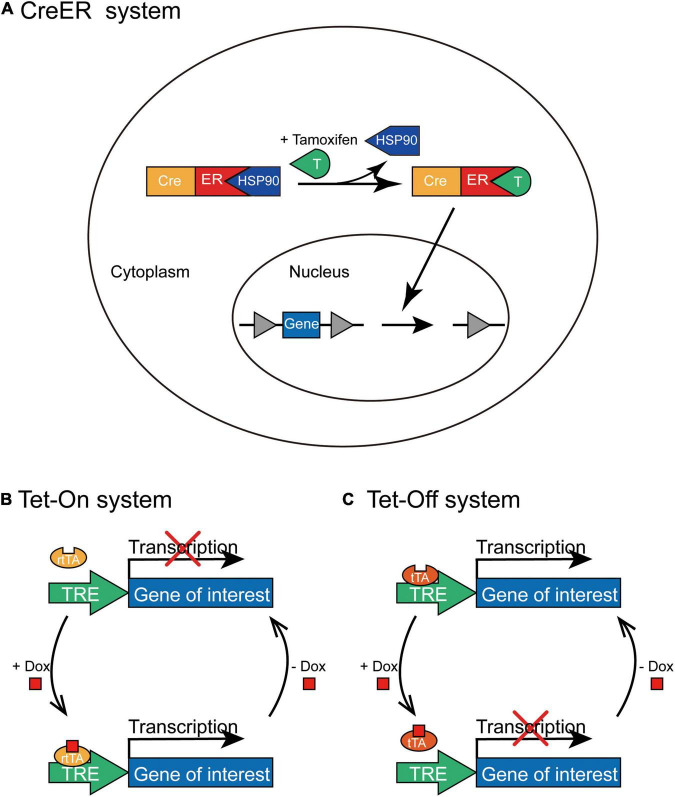
The schematic depiction of CreER and Tet-On/Tet-Off inducible systems. **(A)** In the CreER system, CreERT2 combines with HSP90 without tamoxifen administration and is situated in the cytoplasm. Tamoxifen can replace HSP90 to specifically bind with CreERT2 and lead to the nuclear translocation of CreERT2-tamoxifen complex; thus, Cre-mediated recombination initiates the expression of transgene in the presence of tamoxifen. **(B)** In the Tet-On system, rtTA combines with TRE only in the presence of Dox to activate the transcription of the transgene; however, rtTA cannot combine to the TRE without the administration of Dox. **(C)** In the Tet-Off system, tTA binds to TRE in the absence of Dox to initiate the transcription of the transgene; however, tTA cannot bind to TRE with the administration of Dox.

CreER^*T*2^ obtained by fusing Cre recombinase with the mutated estrogen receptor ER*T^2^* can induce recombination only after the administration of tamoxifen (Feil et al., 2009), but not endogenous estradiol. In the absence of tamoxifen, CreER^*T*2^ interacts with heat shock protein 90 (HSP90) and is sequestered in the cytoplasm. However, tamoxifen administration leads to the dissociation of CreER^*T*2^ and HSP90, which then induces the nuclear translocation of CreER^*T*2^. In the cell nucleus, CreER^*T*2^ recognizes loxP sites to induce recombination; thus, it can control the time window of gene expression by controlling the injection time of tamoxifen (Figure 4A). The CreER system has been widely employed to generate a series of transgenic mouse lines expressing tamoxifen-inducible Cre recombinase (CreER^*T*2^) under the control of different promoters; for example, to label active neurons within a limited time window after tamoxifen administration by expressing CreER^*T*2^ through an activity-dependent IEG promoter (Guenthner et al., 2013).

### Tet-On/Tet-Off System Induced by Doxycycline

This system contains two pivotal components (Gossen and Bujard, 1992): transcriptional transactivators (the reverse tTA (rtTA) and tTA) that interact specifically with the TRE and antibiotics (tetracycline or its derivative, doxycycline) that regulate the bond between tTA/rtTA transactivators and TRE. The expression of the target gene requires rtTA or tTA combined with TRE only in the presence or absence of tetracycline (Tet) or doxycycline (Dox), and the expression of tTA or rtTA transactivators can be further limited to specific cell types. In the Tet-On system, rtTA combines with TRE only in the presence of Dox; therefore, the expression of the gene of interest can be initiated only after Dox is administered at a specific time point (Figure 4B). Whereas, in the Tet-Off system, tTA cannot bind to TRE in the presence of Dox and the transcription of the target gene is squelched (Figure 4C). For example, administration of doxycycline during the memory consolidation phase initiates the expression of shRNA of Suv39h1 through the Tet-On system and then identifies that Suv39h1 knockdown can reduce the stability of established memories (Ding et al., 2017), and the different roles of distinct neuronal ensembles within memory engram have been explored under the temporal control of Tet-Off system (Josselyn and Tonegawa, 2020; Sun et al., 2020).

Therefore, the CreER and Tet-On/Tet-Off systems create an “on/off” switch to express exogenous genes of interest in a transient and reversible manner at a particular time point, gain temporal specificity, and allow the time window of gene expression to be controlled.

## Sparse Labeling

As the basic units of the nervous system, neurons are mutually intertwined to form complex neural networks. Traditional labeling techniques usually map the connections between brain areas or neuronal populations, but are difficult to achieve observation at the single-neuron level. Thus, sparse-labeling systems have been developed that enable to visualize the complete morphology, dissect the connectivity, and interrogate the function of individual cells in densely packed brain regions with tunable sparseness.

Injecting a highly diluted Cre-expressing virus and simultaneously directing a high-titer virus encoding Cre-dependent fluorescent protein allow the generation of sparse but high-intensity labeling of neurons at the injection site and for subsequent reconstruction of individual neurons (Economo et al., 2016; Vrieler et al., 2019). Similar to the intersectional strategy, we can achieve cell type- and/or projection-specific sparse labeling (Han Y. et al., 2018; Zingg et al., 2020).

In the Supernova system, sparse labeling depends on the leaky expression of TRE and bright labeling relies on tTA/TRE positive feedback (Luo et al., 2016). The modified Supernova system that utilizes Cre and FLP as the orthogonal pair contains two AAV vectors: the “controller” vector, which regulates the sparseness of the labeling and includes the TRE promoter and a Cre-dependent expression cassette (DIO) encoding the Flp recombinase, and the “amplifier” vector, which also contains a TRE promoter but carries a Flp-dependent expression cassette (fDIO) encoding fluorescent marker and tTA (Lin et al., 2018). For cell-type-specific sparse labeling, the sparse labeling virus mix is injected into a target area in mice expressing Cre recombinase in a specific cell type. In addition to mapping the projection specificity of a single neuron, a cocktail of two AAV vectors is injected into a target area of interest, and AAV-retro-Cre is delivered to a specific output site.

Transfecting a primary neuron with vectors expressing TVA and RV-G by single-cell electroporation or the whole-cell patch clamp method, followed by infecting the TVA-expressing cells with EnvA-pseudotyped rabies viruses, allows the tracing and genetic targeting of monosynaptic inputs to a single neuron (Marshel et al., 2010; Rancz et al., 2011; Wertz et al., 2015).

## Conclusion

The continuous progress of genetically modified viruses provides us with excellent specificity in how to target and access selective cell types, as well as the functional interrogation and manipulation of particular neuron subpopulations and neural circuits in a high-resolution spatially and temporally specific manner, which has remarkably changed neuroscience research. An ideal engineered viral vector must possess several pivotal properties: large payload, high-titer packaging, low immunogenicity, high cell type-specificity, and tunability in its expression dynamics, which, currently, cannot be achieved simultaneously. AAV has been widely used as the first carrier for circuit mapping and gene therapy, but its limited packing capacity is still a major hindrance. The design and/or discovery of new viral tools that allow for more specific, safer, and stronger transgene expression in targeted cell populations will be pivotal in future research on neural circuits. And using versatile viral tools to dissect how the neural network state changes during dynamical development and in various pathological conditions will also be the focus of future study. Further verification and improvement of the transduction efficiency and long-term safety of viral vectors in non-human primates will pave the way for gene intervention therapies of neurological diseases.

## Author Contributions

YH, XZ, and HZ drafted the manuscript. FL, GY, and JL did figure organization. JZ, XG, and GG proposed the outline and edited the manuscript. All authors contributed to the article and approved the submitted version.

## Conflict of Interest

The authors declare that the research was conducted in the absence of any commercial or financial relationships that could be construed as a potential conflict of interest.

## Publisher’s Note

All claims expressed in this article are solely those of the authors and do not necessarily represent those of their affiliated organizations, or those of the publisher, the editors and the reviewers. Any product that may be evaluated in this article, or claim that may be made by its manufacturer, is not guaranteed or endorsed by the publisher.
